# Activated Macrophages Promote Hepatitis C Virus Entry in a Tumor Necrosis Factor-Dependent Manner

**DOI:** 10.1002/hep.26911

**Published:** 2014-02-25

**Authors:** Nicola F Fletcher, Rupesh Sutaria, Juandy Jo, Amy Barnes, Miroslava Blahova, Luke W Meredith, Francois-Loic Cosset, Stuart M Curbishley, David H Adams, Antonio Bertoletti, Jane A McKeating

**Affiliations:** 1 Hepatitis C Research Group, Institute for Biomedical Research, University of BirminghamBirmingham, UK; 2 NIHR Liver Biomedical Research Unit, University of BirminghamBirmingham, UK; 3 Viral Hepatitis Laboratory, Singapore Institute for Clinical Sciences, Agency of Science Technology and Research (A*STAR)Singapore; 4 Université de LyonLyon, France

## Abstract

Macrophages are critical components of the innate immune response in the liver. Chronic hepatitis C is associated with immune infiltration and the infected liver shows a significant increase in total macrophage numbers; however, their role in the viral life cycle is poorly understood. Activation of blood-derived and intrahepatic macrophages with a panel of Toll-like receptor agonists induce soluble mediators that promote hepatitis C virus (HCV) entry into polarized hepatoma cells. We identified tumor necrosis factor α (TNF-α) as the major cytokine involved in this process. Importantly, this effect was not limited to HCV; TNF-α increased the permissivity of hepatoma cells to infection by Lassa, measles and vesicular stomatitis pseudoviruses. TNF-α induced a relocalization of tight junction protein occludin and increased the lateral diffusion speed of HCV receptor tetraspanin CD81 in polarized HepG2 cells, providing a mechanism for their increased permissivity to support HCV entry. High concentrations of HCV particles could stimulate macrophages to express TNF-α, providing a direct mechanism for the virus to promote infection. *Conclusion*: This study shows a new role for TNF-α to increase virus entry and highlights the potential for HCV to exploit existing innate immune responses in the liver to promote *de novo* infection events. (Hepatology 2014;59:1320-1330)

Hepatitis C virus (HCV) is a member of the flaviviridae family and an important human pathogen that infects hepatocytes leading to progressive liver disease including fibrosis, cirrhosis, and hepatocellular carcinoma. A number of antivirals targeting the HCV replicase are in development; however, recent trials show reduced efficacy in patients with cirrhosis or comorbidities, genotype-dependent differences in drug sensitivity, and the emergence of drug-resistant viruses.^1^,^2^ HCV entry into host cells provides a conserved target for therapeutic intervention and the potential for combination therapy with anti-replicase drugs.^1^ HCV entry into the host is mediated by molecules or receptors at the cell surface and current evidence supports a multistep process involving scavenger receptor class B member I, tetraspanin CD81, and tight junction proteins claudin-1 and occludin in HCV internalization (reviewed^3^,^4^). Recent studies have shown the limited ability of polarized hepatocytes to support HCV entry,^5^,^6^ highlighting a need to understand the physiological processes that regulate hepatocellular permeability in the infected liver.

Inflammation is associated with chronic HCV infection and drives liver injury.^7^ However, with the possible exception of interferons, the role of proinflammatory mediators in the HCV lifecycle is poorly understood. Macrophages play an essential and early role in sensing pathogens by way of expression of Toll-like receptors (TLRs) that recognize pathogen-associated molecular patterns that prime cytokine expression, mainly interleukin (IL)−1β, IL-6, and tumor necrosis factor alpha (TNF-α).^8^ Kupffer cells (KCs) are the resident macrophage of the liver and in addition to early innate sensing of pathogens help to maintain the tolerogenic environment of the liver.^9^ The chronic HCV-infected liver shows a significant increase in total macrophage numbers that most likely represents a local proliferation of KCs and infiltration of bone-marrow-derived monocytes^10^; however, their role in the HCV lifecycle is poorly understood. We demonstrate that activation of blood-derived and intrahepatic macrophages with a panel of TLR agonists induce soluble mediators that promote HCV entry into polarized hepatoma cells. We identify TNF-α as the major cytokine involved in this process. The ability of TNF-α to promote the permissivity of polarized hepatoma cells was not limited to HCV but was seen with lentiviral pseudotypes expressing Lassa, measles, and VSV-G glycoproteins. These studies identify a new pathway for HCV to exploit inflammatory responses in the chronically infected liver to promote infection that may contribute to viral persistence and provide new targets for therapeutic intervention.

## Materials and Methods

### Cells and Reagents

HepG2 (American Tissue Culture Collection, Rockville, MD [ATCC]) were maintained in Dulbecco's modified Eagle's medium (DMEM) with 10% fetal bovine serum. HepG2.CD81 were generated by lentiviral transduction as described, supplemented with 400 μg/mL Zeocin^5^ and polarized by seeding at 1 × 10^5^cells/cm^2^ and culturing for 5 days.^5^ KCs were isolated from surplus liver samples from the liver transplant program at the Queen Elizabeth Hospital, Birmingham, UK. Peripheral CD14^+^ monocytes were isolated from healthy donors and stimulated as described in the Supporting Information.^13^ Human liver-associated mononuclear cells were collected from liver washouts, surface-stained for monocyte and lineage-positive markers, stained for TNF-α / interferon-gamma (IFN-γ) and cells characterized on a BD LSRII flow cytometer and analyzed with FACS Diva software.

### Pseudoparticle Genesis and Infection

Pseudoparticles were generated by transfecting 293T cells with plasmids encoding a human immunodeficiency virus (HIV) provirus expressing luciferase and viral envelope glycoproteins generated from HCV (strain H77), Lassa, measles, and VSV-G^14^ or no-envelope control.^15^ Supernatants were harvested 48 hours posttransfection and stored at −80°C prior to infection of target cells.

### HCVcc Genesis and Infection

HCV SA13/JFH was generated and infection quantified by enumerating NS5A-positive foci and real-time polymerase chain reaction (PCR) detection of viral RNA as described in the Supporting Information. HCV SA13/JFH or mock-electroporated Huh-7.5 supernatants were collected, concentrated through a Vivaspin 100,000 MWCO concentrator, overlaid on 20% sucrose, and centrifuged for 24 hours at 23,000 rpm to purify virus particles. Virus-containing and mock-infected supernatants were concentrated equally.

### Viral Infection Studies

HepG2.CD81 cells were seeded at 1 × 10^5^ cells/cm^2^ and allowed to polarize for 5 days.^5^ Cells were treated with conditioned medium (CM) from peripheral macrophages, dendritic cells (DCs), or liver-derived KCs or recombinant human cytokines, and pseudoparticles or HCVcc added immediately or after one 1 hour and the cultures incubated for 72 hours. To neutralize TNF activity, CM or recombinant cytokines were incubated for 1 hour with anti-TNF (Infliximab; Merck; 10 μg/mL) prior to adding to HepG2.CD81 cells. After 72 hours cells were lysed (Promega, Madison, WI) and luciferase activity measured. Relative infectivity was calculated by subtracting the no-envelope signal and expressing the viral signal relative to the untreated control. To quantify HCVcc infection, viral RNA was measured using real-time PCR and HCV antigen expressing cells enumerated by indirect immunofluorescent detection of NS5A.

### Quantification of HepG2 Permeability

To determine the functionality of bile-canalicular (BC) tight junctions, HepG2 cells were incubated with 5 μM 5-chloromethylfluorescein diacetate (CMFDA; Invitrogen) at 37°C for 10 minutes to allow translocation to BC lumen. After extensive washing, the capacity of BC to retain CMFDA was enumerated using a fluorescence microscope.

### Confocal Imaging of Occludin Localization

HepG2.CD81 cells were grown on 13 mm glass coverslips (Fisher Scientific) for 5 days and fixed with ice-cold methanol and occludin visualized by staining with 2 μg/mL mouse anti-occludin (Invitrogen) and antirabbit Alexa-Fluor 488 (Invitrogen). Nuclei were counterstained with 4′,6-diamidino-2-phenylindole (DAPI) and mounted with ProLong Gold (Invitrogen). Cells were viewed by laser-scanning confocal microscopy on a Zeiss META head microscope with a 100× oil-immersion objective.

### Fluorescent Recovery After Photobleaching (FRAP)

HepG2 cells were transduced with AcGFP-tagged CD81^16^ and allowed to polarize for 5 days on glass-bottomed plates. Cells were treated for 1 hour with TNF-α (100 ng/mL) and FRAP performed on a Zeiss LSM78 confocal microscope as previously described.^16^ Data were imported into GraphPad Prism and fitted using a single exponential decay algorithm, Y=Span(1-exp(-K*X))+plateau. The diffusion coefficient (D) was calculated using a 2D diffusion model for a circular bleached region of interest, where D = 0.224 × (radius^2^/t_1/2_).

### Statistical Analysis

Statistical analyses were performed using Student *t* test in Prism 6.0 (GraphPad, San Diego, CA) with *P* < 0.05 being considered statistically significant.

## Results

### Activated Macrophages Promote HCV Entry

**Fig 1:**
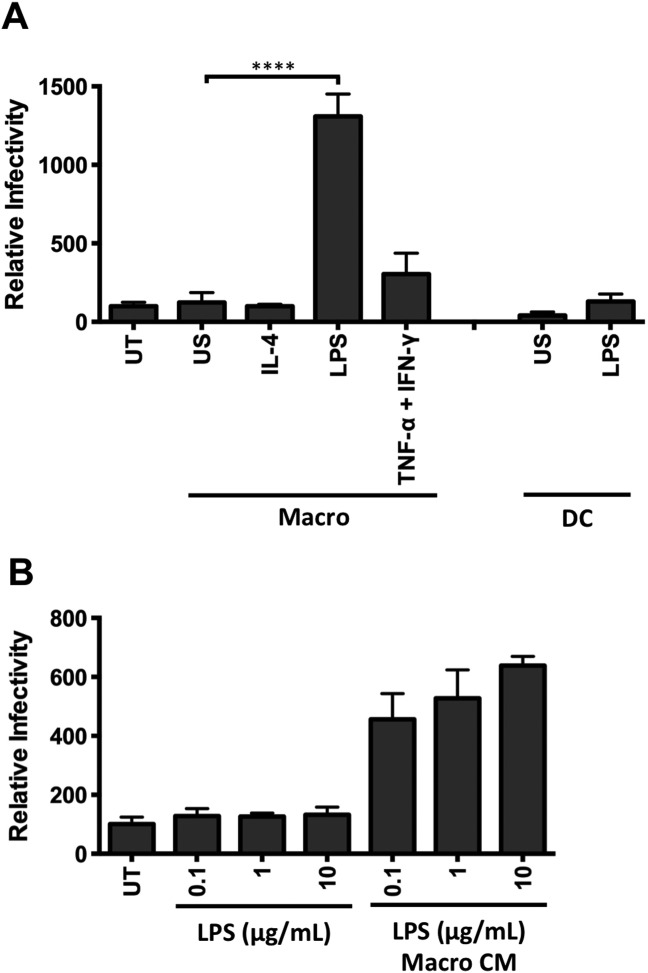
Conditioned media from LPS-stimulated macrophages promotes HCV entry. (A) Primary macrophages (Macro) and dendritic cells (DC) isolated from CD14^+^ peripheral monocytes from the same donor were stimulated with IL-4 (10 ng/mL), LPS (1 μg/mL), TNF-α/IFN-γ (10 ng/mL), or unstimulated (US) for 24 hours and conditioned media (CM) harvested. Polarized HepG2.CD81 cells were treated with CM at a final 1:2 dilution and infected with HCVpp. (B) Polarized HepG2-CD81 cells were incubated with LPS alone or CM from LPS-stimulated macrophages (Macro CM) and infected with HCVpp. Data are presented as mean infectivity ± SD relative to the untreated control (UT) (n = 5 independent experiments). ****P < 0.0001, **P < 0.01.

To assess the role of macrophages in the HCV lifecycle, peripheral blood-derived CD14^+^ monocytes were cultured on plastic and stimulated with a combination of IFN-γ and TNF-α to generate M1-macrophages, IL-4 to generate M2-macrophages, or activated with lipopolysaccharide (LPS) for 24 hours. DCs were isolated from the same donor and stimulated with LPS to stimulate the liver microenvironment. CM was collected and assessed for its effect on HCV pseudoparticle (HCVpp) infection of polarized HepG2.CD81 cells. CM from LPS-stimulated macrophages, but not DCs, significantly increased HCVpp infection, whereas LPS alone had no effect (Fig. 1A). In contrast, IL-4 or TNF-α/IFN-γ stimulated macrophage CM had no effect on HCVpp infectivity (Fig. 1A). Macrophages stimulated with a range of LPS concentrations (0.1-10 μg/mL) increased HCVpp infection (Fig. 1B) and this was consistent between donors (data not shown). These experiments show that LPS-stimulated monocyte-derived macrophages secrete soluble factors that promote HCVpp entry.

### IL-1β and TNF-α Enhance HCV Infection

**Fig 2:**
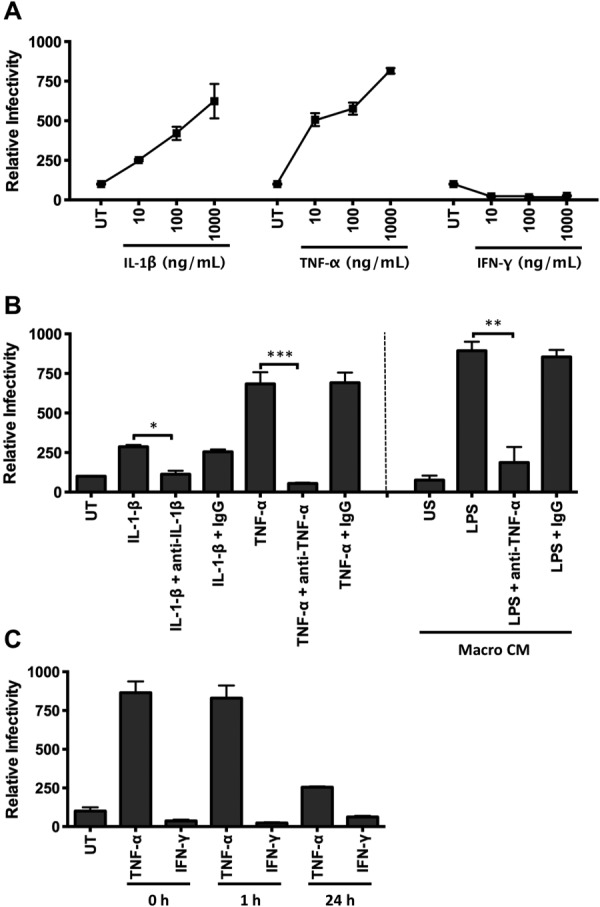
TNF-α is the major factor secreted from activated macrophages that promotes HCV infection. (A) Polarized HepG2.CD81 cells were treated with IL-1β, TNF-α, or IFN-γ and infected with HCVpp. (B) Neutralization of IL-1β, TNF-α, and LPS-stimulated macrophage CM (Macro CM) proviral effect with anti-IL-1β (10 μg/mL) or anti-TNF-α (10 μg/mL). (C) HepG2.CD81 cells were pretreated with TNF-α (10 ng/mL) and IFN-γ (100 ng/mL) for 1 or 24 hours prior to inoculating with virus or at the same time (0 hours). Data are presented as mean ± SD relative to the untreated control (UT) (n = 3 independent experiments). ***P < 0.001, **P < 0.01.

To identify the proviral factors expressed by macrophages following LPS stimulation we quantified the expression of 84 cytokines by PCR gene array from untreated, IL-4, or LPS-stimulated macrophages and DCs. We observed a different profile of cytokine messenger RNA (mRNA) expression in macrophages stimulated with IL-4 or LPS (Supporting Fig. 1). LPS stimulated macrophages to express high levels of TNF-α and low levels of IL-1β, whereas DCs expressed several interferons (IFN-α, IFN-β, and IFN-γ) along with TNF-α (Supporting Figs. 1, 2). To investigate which proinflammatory cytokines promote HCVpp infection we screened a panel of cytokines that were shown to be upregulated in the array including IL-1β, IL-6, IL-8, IL-10, IL-17, IL-18, TNF-α, and IFN-γ (Supporting Fig. 1). Both IL-1β and TNF-α increased HCVpp (strain H77) infection of polarized HepG2.CD81 cells, whereas IFN-γ reduced infection in a dose-dependent manner (Fig. 2A). TNF-α promoted infection of HCVpp expressing a diverse panel of patient-derived envelope glycoproteins (data not shown). Furthermore, combining IL-1β or TNF-α with IFN-γ (100 ng/mL) reduced infection to control levels, similar to that of IFN-γ alone (data not shown). Replication of the lentiviral genome following lipid-based delivery directly into HepG2.CD81 was not modulated by cytokine treatment (data not shown), demonstrating a specific effect of the cytokines on HCV glycoprotein-dependent particle entry.

We confirmed that anti-TNF-α and anti-IL-1β neutralizing antibodies inhibited the effect of recombinant TNF-α and IL-1β on HCVpp infection (Fig. 2B). Importantly, preincubation of LPS-stimulated macrophage CM with anti-TNF-α reduced HCVpp infection to a comparable level observed with untreated hepatoma cells. The additional observation that LPS stimulates macrophages to secrete high levels of TNF-α (Supporting Fig. 2) supports a key role for this cytokine in promoting HCV infection (Fig. 2B). In contrast, LPS-stimulated DCs secrete both TNF-α and IFN-γ (Supporting Fig. 2), which may explain the modest effect of their CM on HCVpp entry (Fig. 1), suggesting a balance between the antiviral activity of IFN-γ and proviral effect of TNF-α. Pretreating polarized hepatoma cells with TNF-α for 1 hour prior to inoculating with virus led to a significant increase in infection; however, a longer preincubation time of 24 hours ablated the cytokine mediated effect on HCVpp infection, suggesting a limited response time for the cells and/or a reversible phenotype (Fig. 2C). These studies uncover a new role for activated macrophage expressed TNF-α to promote HCV entry into polarized hepatoma cells.

**Fig 3:**
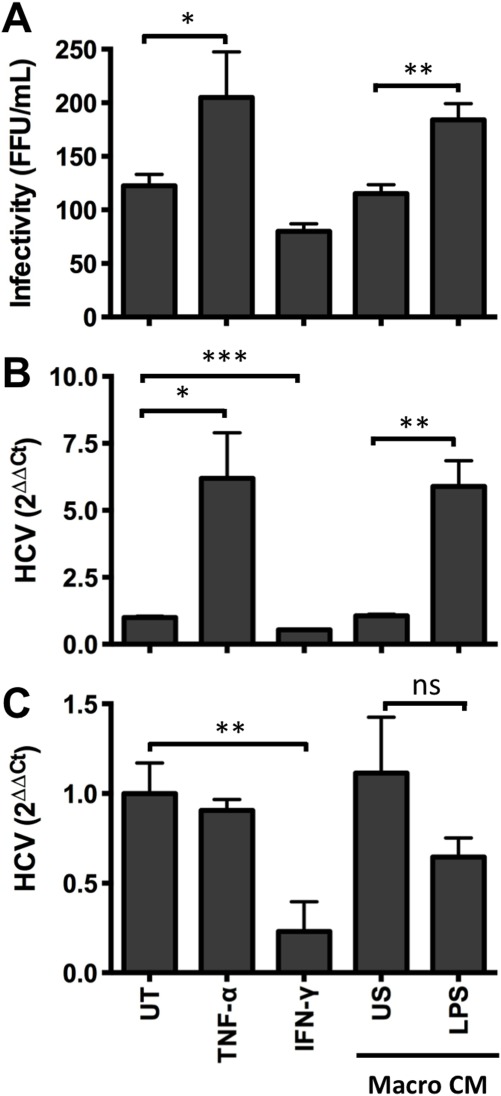
Cytokines increase HCVcc infection. HepG2.CD81 were treated with cytokines or CM from unstimulated (US) or LPS-stimulated macrophages and infected with HCV (SA13/JFH) for 12 hours. After 72 hours NS5A+ cells (A) and RNA levels (B) were quantified. (C) To assess the effect of cytokines on viral replication, HepG2 cells stably supporting HCV-JFH-1 (HepG2-JB) were treated with cytokines or CM and HCV RNA quantified. Data are presented as mean ± SD relative to the untreated control (UT) (n = 3 independent experiments). ***P < 0.001, **P < 0.01, *P < 0.05.

To ascertain whether TNF-α promotes infection of authentic HCV particles, polarized HepG2.CD81 were treated with recombinant cytokines or CM from LPS-stimulated macrophages for 1 hour and infected with HCV strain SA13/JFH-1 or JFH-1. Infection was quantified by measuring the frequency of NS5A antigen-expressing cells by indirect immunofluorescence and qRT-PCR of HCV RNA. TNF-α stimulated a significant increase in the number of HCV-infected HepG2.CD81 cells; however, this effect was less pronounced compared to the increase in viral RNA (Fig. 3A,B). To assess whether TNF-α stimulated viral replication, HepG2 cells stably replicating a blasticidin-tagged HCV-JFH1 full-length^17^ were treated with TNF-α or CM from LPS-stimulated macrophages for 3 or 24 hours. There was no significant increase in HCV RNA levels following treatment with TNF-α or CM. In contrast, IFN-γ reduced the level of viral RNA (Fig. 3C). These experiments illustrate the role of TNF-α in promoting the entry of authentic HCV particles with negligible effect on genomic RNA replication.

### Diverse TLR Agonists Stimulate Macrophages to Promote HCV Infection

**Fig 4:**
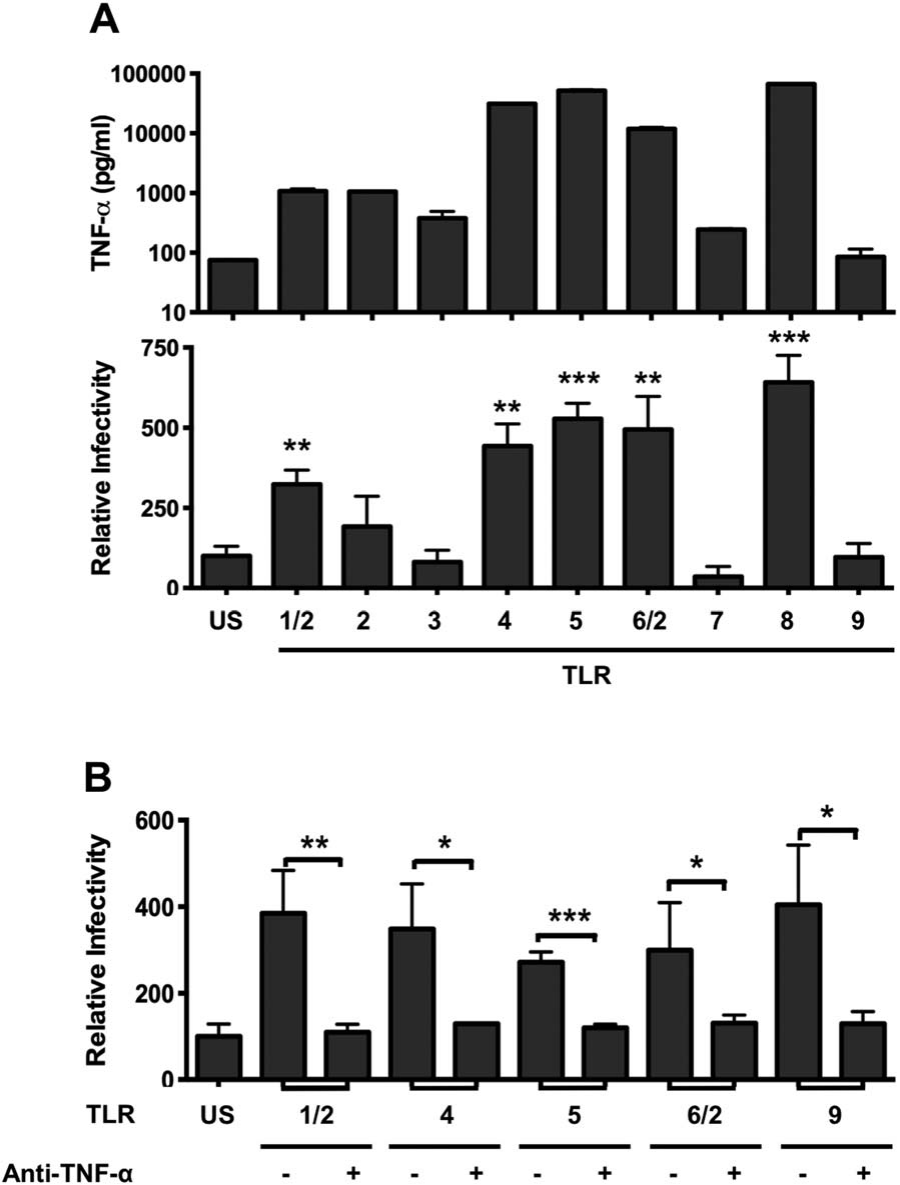
TLR-stimulated macrophages express TNF-α that promotes HCV entry. (A) Peripheral macrophages were stimulated with TLR agonists: Pam3CSK4 (TLR1/2)1 μg/mL; HKLM (TLR2) 10^8^ cells/mL; PolyI:C (TLR3) 10 μg/mL; LPS (TLR4)10 μg/mL; flagellin (TLR5) 10 μg/mL; FSL-1 (TLR6/2)1 μg/mL; imiquimod (TLR7)10 μg/mL; ssRNA40 (TLR8) 10 μg/mL; ODN2006 (TLR9) 5 μM for 24 hours and CM assayed for TNF-α (upper graph) and effect on HCVpp infection of polarized HepG2.CD81 cells (lower graph). (C) TLR-stimulated macrophage CM was preincubated with anti-TNF-α (10 μg/mL) for 1 hour and evaluated for its effect on HCVpp infection of polarized HepG2.CD81. Data are presented as mean ± SD relative to unstimulated (US) control (n = 3 independent experiments). ***P < 0.001, **P < 0.01, *P < 0.05.

To date, 10 functional TLRs have been identified in humans (TLR1-10) that detect specific pathogen-associated molecular patterns. To investigate whether diverse TLR agonists stimulate macrophages to express TNF-α and promote HCVpp infection, blood-derived macrophages were stimulated with agonists for 24 hours and CM assessed for its effect on HCVpp infection. Treating hepatoma cells with agonists had no effect on HCVpp infection (data not shown). All TLR agonists, with the exception of CpG (TLR9), stimulated macrophages to express varying levels of TNF-α (Fig. 4A) that correlated with their ability to promote HCVpp infection (R^2^ = 0.7313) (Fig. 4A). Furthermore, the enhancing effect of the various CM could be neutralized with anti-TNF (Fig. 4B). LPS, flagellin, and ssRNA-40 stimulated macrophages to secrete low levels of IL-1β; however the levels were insufficient to promote HCVpp infection (Supporting Fig. 2). Taken together, these data demonstrate that TNF-α is the major cytokine produced by macrophages stimulated with a variety of bacterial and viral TLRs that promotes HCV infection.

### TNF-α Promotes Entry of Diverse Viruses Into Polarized Hepatocytes

**Fig 5:**
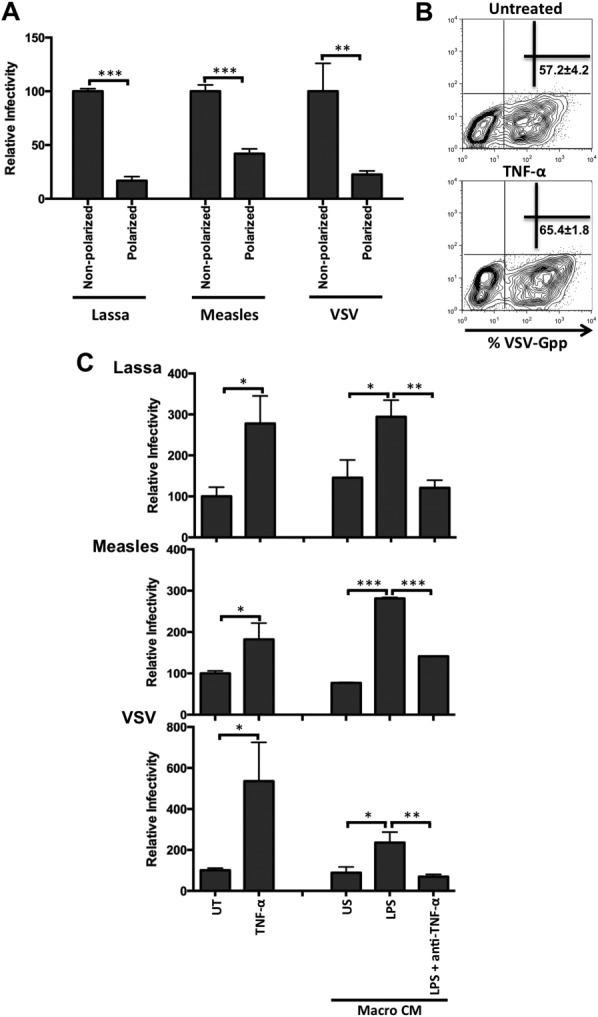
Cytokines increase the permissivity of polarized hepatocytes to diverse viral pseudoparticles. (A) Lassa, measles, and vesicular stomatitis virus pseudoparticle infection of polarized and nonpolarized HepG2.CD81 cells. (B) Polarized HepG2.CD81 were infected with GFP-tagged VSV-Gpp ± TNF-α (100 ng/mL) and infected cells quantified by flow cytometry. Mean ± SD percent of GFP^+^ infected cells are shown. (C) Lassa, measles, and VSV-G pseudoparticle infection of HepG2.CD81 cells treated with TNF-α (10 ng/mL) or CM from unstimulated (US) or LPS-stimulated macrophages with or without anti-TNF-α (10 μg/mL). Data are presented as mean ± SD relative to untreated control (UT) (n = 3 independent experiments). ***P < 0.001, **P < 0.01, *P < 0.05.

To determine whether the proviral effect of TNF-α is specific for HCV, we generated pseudoparticles bearing the surface glycoproteins of Lassa, measles, and vesicular stomatitis virus (VSV). HepG2 polarization restricted the infection of all pseudoparticles tested (Fig. 5A). To determine whether TNF-α increased the number of infected cells or the viral burden per cell, we generated VSV-G pseudoparticles expressing a fluorescent reporter protein. Flow cytometry revealed that TNF-α increased both the number of infected cells (57% ± 4.2% versus 65% ± 1.8%) and their fluorescent intensity (Fig. 5B). CM from LPS-stimulated macrophages increased the permissivity of HepG2.CD81 cells to all pseudoparticles in a TNF-dependent manner (Fig. 5C). These results highlight the role of TNF-α in enhancing the permissivity of polarized HepG2 cells to support infection by diverse pseudoparticles.

### TNF-α Increases Hepatoma Permeability

**Fig 6:**
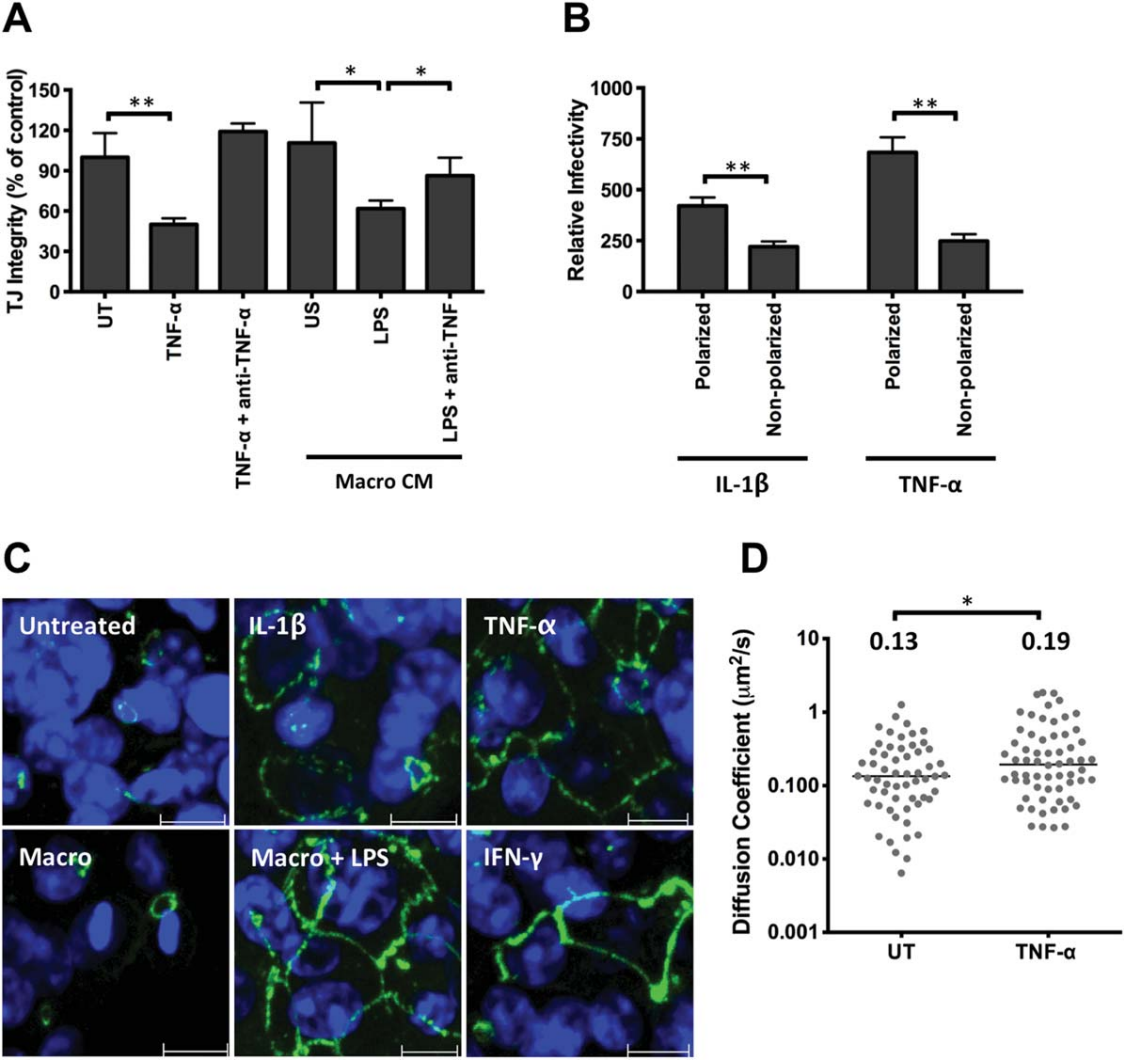
Effect of TNF-α on HepG2 permeability, occludin localization, and CD81 lateral diffusion. (A) Polarized HepG2.CD81 were treated with cytokines or CM from unstimulated (US) or LPS-stimulated macrophages, with or without anti-TNF-α (100 μg/mL) and tight junction integrity quantified. (B) Effect of IL-1β or TNF-α on HCVpp infection of polarized or nonpolarized HepG2.CD81. Data are expressed relative to untreated (100%) polarized or nonpolarized control. (C) Occludin localization in HepG2.CD81 cells following cytokine or CM treatment, where scale bar = 10 μm. (D) AcGFP-CD81 diffusion speed in untreated (UT) or TNF-α treated polarized HepG2 cells, where each symbol represents an independent bleach spot. The median values are shown. **P < 0.01, *P < 0.05.

Proinflammatory cytokines are known to disrupt epithelial permeability; however, this is largely based on gut intestinal cell models and there is limited information for hepatocytes.^18^ We demonstrate that TNF-α and CM from LPS-stimulated macrophages disrupted tight junction integrity, where the latter was neutralized by anti-TNF antibody (Fig. 6A), suggesting that TNF-α promotes HCVpp infection by increasing HepG2 permeability. Indeed, TNF-α and IL-1β increased the permissivity of polarized, but not nonpolarized, HepG2.CD81 cells, supporting this conclusion (Fig. 6B). In addition, there was minimal effect of TNF-α or IL-1β on the permissivity of Huh-7 or Huh-7.5 hepatoma cells to support HCVpp entry, which do not polarize in vitro.^5^

HepG2 cells express the tight junction protein occludin at BC structures, representing complex hepatocellular polarity. Treatment with TNF-α, IL-1β, or CM from LPS-stimulated macrophages induced a redistribution of occludin to the basolateral membrane of HepG2.CD81 cells (Fig. 6C). In contrast, the cytoplasmic junctional protein ZO-1 was unaffected (data not shown), supporting cytokine disruption of tight junctions. We previously reported that polarization reduced viral receptor CD81 and HCVpp diffusion at the basal HepG2 membrane, which may explain their reduced permissivity.^16^ To investigate the effect of TNF-α on CD81 dynamics, HepG2 were transduced to express AcGFP-CD81 and lateral diffusion at the basal membrane measured by FRAP. TNF-α increased CD81 diffusion coefficient (Fig. 6D). Together, these data highlight a role for TNF-α in regulating hepatoma tight junction and CD81 dynamics, providing a mechanism for the increase in viral permissivity.

### HCV and LPS Stimulate Kupffer Cells to Express TNF-α

**Fig 7:**
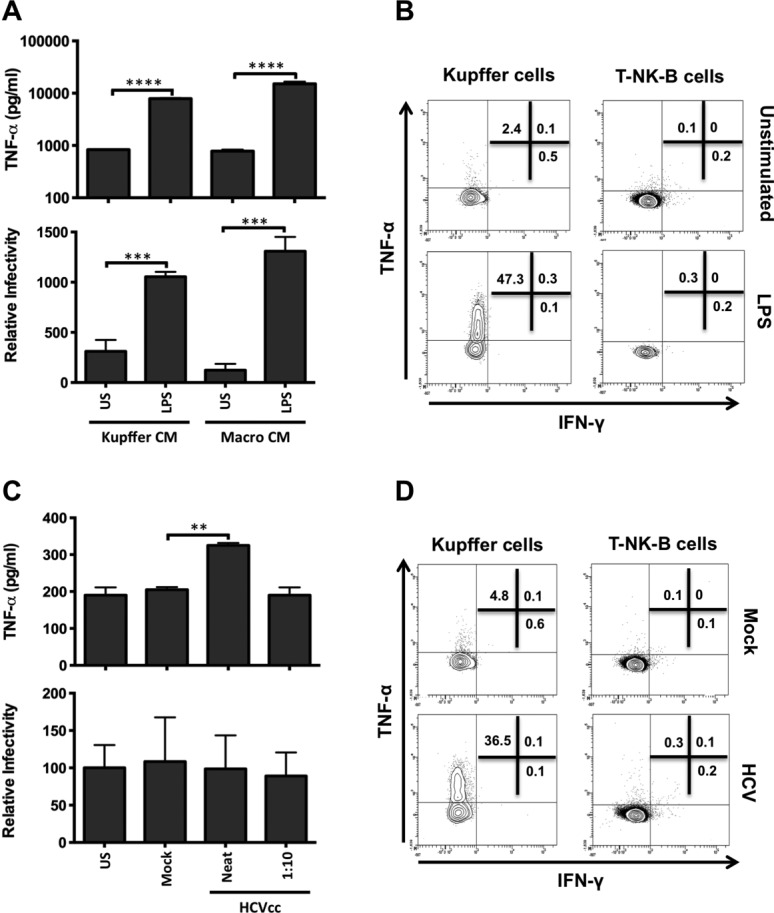
Kupffer cells express TNF-α but not IFN-γ following LPS and HCV stimulation. (A) Intrahepatic KCs or peripheral blood-derived macrophages were stimulated for 24 hours with LPS (10 μg/mL), CM collected and assayed for TNF-α (upper graph), and effect on HCVpp infection of polarized HepG2.CD81 cells (lower graph). (B) Intrasinusoidal monocytes and lineage-positive (T-NK-B) cells were stimulated with LPS (10 μg/mL) for 6 hours and intracellular TNF-α and IFN-γ expression assessed by flow cytometry. (C) Intrahepatic KCs or peripheral blood-derived macrophages were incubated with HCV (strain SA13/JFH) or mock at an estimated multiplicity of 22 (Neat) or 2.2 (1:10), as defined by HCV-RNA copies/cell. CM was collected and assayed for TNF-α (upper graph) and effect on HCVpp infection of polarized HepG2.CD81 cells (lower graph). (D) Intrasinusoidal monocytes and lineage-positive (T-NK-B) cells were stimulated with HCV (neat) or mock antigen for 6 hours and intracellular TNF-α and IFN-γ expression assessed by flow cytometry. Data are represented as mean ± SD (n = 2 independent experiments). ***P < 0.001, **P < 0.01, *P < 0.05.

Liver resident macrophages or KCs are reported to be tolerant to LPS. We therefore compared the sensitivity of KCs and peripheral blood-derived macrophages to LPS stimulation. KCs and peripheral-derived cells responded to LPS and expressed comparable levels of TNF-α and increased HCVpp infection (Fig. 7). To investigate the effect of LPS on a mixed liver immune cell population, we studied the responsiveness of cells harvested by perfusing normal healthy liver donor livers prior to organ transplantation. KCs express TNF-α but no IFN-γ following LPS stimulation, whereas natural killer (NK), T, or B cells from the same donor failed to express detectable levels of TNF-α (Fig. 7B). HCV was recently reported to activate the inflammasome and to promote macrophage secretion of IL-1β.^19^ To ascertain whether HCV could stimulate macrophages to express TNF-α we incubated the sinusoidal immune cell population and blood-derived macrophages with purified virus for 24 hours. Intracellular cytokine staining showed that HCV induced sinusoidal KCs to express TNF-α, whereas mock treatment had no effect (Fig. 7D). High concentrations of HCVcc particles stimulate macrophages to secrete TNF-α that had no detectable effect on HCVpp entry (Fig. 7B), implying that the levels of biologically active TNF-α are too low to be effective or that the virus stimulates macrophages to express additional antiviral mediators. Taken together, these data highlight a role for HCV particles and bacterial products in the liver environment to induce a proinflammatory environment that promotes HCV infection.

## Discussion

We describe a new mechanism for HCV to use TNF-α to promote infection of polarized hepatocytes. Several TLR agonists including LPS stimulate macrophages to secrete cytokines including IL-1β and TNF-α that promote HCV infection. In contrast, LPS activates DCs to secrete high levels of IFN-γ,^20^ a cytokine with antiviral activity, explaining the negligible effect of DC conditioned media on HCV entry. Given the constant stimulation of hepatic TLR4 with gut-derived LPS the liver is thought to have developed tolerance mechanisms to limit hyperactivation of the immune system.^21^ However, recent studies demonstrate a loss of TLR tolerance in macrophages from chronic hepatitis B and C-infected patients, reporting an association between LPS-induced macrophage activation and progression to endstage liver disease.^22^,^23^ It is interesting to note that alcohol^24^ and HIV coinfection^25^ are both associated with increased levels of plasma LPS. Our demonstration that LPS stimulates KCs to promote HCV infection provides a potential explanation for how these comorbidities may augment HCV infection and ensuing liver disease.

The major cell type within the liver supporting HCV replication is the polarized hepatocyte. HCV enters hepatocytes by way of a multistep process that involves two tight junction proteins, claudin-1 and occludin, that reside within BC structures that are poorly accessible to virus entering the liver by way of the sinusoidal blood. This model of virus internalization by way of the hepatocellular tight junction^26^ is consistent with our earlier observation that hepatoma polarization restricts HCV entry. Our recent report that hepatoma polarization reduces CD81 and HCVpp dynamics at the basal membrane compared to nonpolarized cells^16^ highlights a role for membrane protein dynamics in limiting HCV infection of polarized epithelia. Inflammatory cytokines including TNF-α permeabilize epithelia and increase the exposure of underlying tissues to luminal antigens in sites such as the gut and airway, resulting in a multitude of disease states including inflammatory bowel disease, asthma, and cystic fibrosis.^27^,^28^ TNF-α disrupted HepG2 tight junction integrity with a concomitant relocalization of occludin and increase in CD81 dynamics.^16^,^29^ These data are consistent with reports of TNF-α inducing tight junction disassembly in epithelia by way of NF-κB-dependent activation of myosin light chain kinase (MLCK) transcription and MLC phosphorylation.^30^,^31^ We did not observe any effect of TNF-α on HCV replication, suggesting that increased virus entry does not lead to quicker sensing of HCV and reduction of viral replication. Our observation that TNF-α promotes the entry of several pseudoviruses to polarized hepatoma cells suggests that TNF-α affects the function or distribution of several different classes of viral receptors, most likely through modulating membrane receptor trafficking.

Adenovirus was recently shown to activate macrophages to express IL-8, leading to a relocalization of basolateral and junctional expressed viral receptors, αvβ3-5 integrins and CAR, respectively, to the apical surface increasing virus binding and internalization.^32^ We demonstrate that HCV can activate macrophages to express TNF-α; however, the level of secreted TNF-α was insufficient to prime infection and was significantly lower than observed with other TLR agonists. Negash et al.^19^ recently reported that HCV can activate the inflammasome and promote macrophages to secrete IL-1β. We confirm these observations; however, the levels of IL-1β were low and had minimal effect on HCVpp infection. Several publications report that HCV induces TNF-α and IL-1β expression in hepatocytes,^33^,^34^ providing an additional pathway for HCV to promote cytokine expression. These data highlight the potential for HCV to exploit preexisting inflammatory responses to prime viral infection and transmission within the liver.

We used a therapeutic anti-TNF-α monoclonal antibody (infliximab) to neutralize the effect of LPS-stimulated macrophages on viral infection. This and similar antibodies have been used to treat HCV-infected patients with chronic inflammatory conditions such as rheumatoid arthritis and Crohn's disease.^35^ One phase II trial reported that the anti-TNF-α antibody etanercept in combination with interferon and ribavirin significantly reduced HCV replication, with undetectable levels of HCV RNA in 63% of etanercept-treated patients after 24 weeks, compared with 32% of patients receiving interferon and ribavirin therapy.^36^ Despite the well-recognized role of TNF-α in liver injury it has an equally important function in immune protection, highlighted by the enhanced susceptibility of patients with alcoholic liver disease to infectious agents. TLR antagonists have been used to treat a number of diseases^37^ and our study highlights a potential role for TLR4 antagonists to treat viral hepatitis.^38^

In conclusion, we demonstrate a new pathway for HCV to exploit macrophage-expressed TNF-α to promote infection. TNF-α plays a key role in regulating cell differentiation, proliferation, innate and adaptive immune responses, and is expressed by a variety of immune cells. TNF gene polymorphism has been reported to influence serum levels of TNF and to affect susceptibility to various diseases.^39^ Our studies uncover a new role for TNF-α and IL-1β in promoting HCV infection of polarized hepatocytes and provide new therapeutic targets for antiviral therapy.

## Abbreviations

BC: bile canaliculus
CM: conditioned medium
HCV: hepatitis C virus
IFN: interferon
LPS: lipopolysaccharide
PAMP: pathogen-associated molecular pattern
SR-BI: scavenger receptor B-1
TLR: Toll-like receptor
TNF: tumor necrosis factor
VSV: vesicular stomatitis virus
