# Supplementary material for: Activated Macrophages Promote Hepatitis C Virus Entry in a Tumor Necrosis Factor-Dependent Manner
Source: Hepatology. 2014 Feb 25;59(4):1320–30. doi: 10.1002/hep.26911 (PMC4255687; doi:10.1002/hep.26911)

Supplementary Information.

Isolation of Kupffer cells and peripheral monocytes. Kupffer cells were isolated as previously described (1). Briefly, surplus liver specimens from the liver transplant program at the Queen Elizabeth Hospital, Birmingham, were homogenised and digested in Gey's balanced salt solution and layered on Nycodenz (Axis-Shield) gradient before being cultured in RPMI-1640 medium with 10% heat-inactivated human serum. Informed consent in writing was obtained from each patient and the use of material was approved by the University of Birmingham institutional review committee. No donor organs were obtained from institutionalized persons. Kupffer cell phenotype was confirmed by expression of CD68, CD14, CD16, CD11b and CD32 and induction of CD163 in response to LPS stimulation and CD206 in response to IL-4. Peripheral $CD14^+$ monocytes from healthy donors were isolated by density-gradient centrifugation. $CD14^+$ cells were positively selected using a Miltenyi Biotec LS column/AutoMACS Pro separator. To differentiate $CD14^+$ monocytes into macrophages, the cells were cultured on plastic for 5 – 7 days. Macrophage differentiation was confirmed by flow cytometric analysis of CD14, CD16, CD163 or CD206 expression. In parallel, $CD14^+$ cells from the same donor were differentiated into DCs with 1000 IU/ml each of GM-CSF/IL-4 and cells confirmed to express HLA-DR, CD80, CD86, CD11c and low level CD68/CD206 staining. Cells were stimulated for 24h with 10ng/mL IFN-γ/TNF-α or IL-4, or agonists to stimulate TLR 1-9 (Invivogen): Pam3CSK4 (TLR1/2) 1µg/mL, HKLM (TLR2) 10^8cells/mL, PolyI:C (TLR3) 10µg/mL, LPS (TLR4) 10µg/mL, Flagellin (TLR5) 10µg/mL, FSL-1 (TLR6/2) 1µg/mL, Imiquimod (TLR7) 10µg/mL, ssRNA40 (TLR8) 10µg/mL, ODN2006 (TLR9) 5µM for 24h.

Human liver-associated mononuclear cells were collected from liver wash-outs with patient consent and institutional human ethics approval (Parkway IEC approval 2012/037)(2). $CD14^+$ cells (negative for CD3, CD7, CD56, CD19 and CD20) expressed CD68 and CD11b and were defined as Kupffer cells. The total cell population purified from the liver wash-out were plated in a 96-well round bottom plate, treated with Brefeldin A (10ug/ml) and stimulated with LPS (1ug/ml) for 6h. Human liver-associated mononuclear cells were surface-stained for monocyte

and CD14 and CD16 (for monocytes), CD3, CD7, CD56, CD19 and CD20 for lineage-positive cells, i.e. T/NK/B cells.

Primary antibodies used were: anti-HCV NS5A-9E10 (C. Rice, Rockefeller University) and rabbit anti-occludin (Invitrogen). Immunoglobulin from normal healthy volunteers and chronically HCV infected donors was purified by Protein-G affinity chromatography. Fluorescent-labelled secondary antibodies, Alexa Fluor 488 anti-mouse and anti-rabbit IgG were obtained from Invitrogen. Recombinant cytokines were from Peprotech.

Quantification of tight junction integrity. Tight junction integrity in polarized HepG2.CD81 cells was quantified as previously described (3, 4). Briefly, HepG2 cells were washed with PBS and incubated with 5 µM 5-chloromethylfluorescein diacetate (CMFDA; Invitrogen) at 37°C for 10 min to allow internalization and translocation to the BC lumen by MRP2. After washing with PBS extensively, the capacity of BC lumens to retain CMFDA was analyzed using a fluorescence microscope.

HCVcc generation. Plasmids encoding HCV strains J6/JFH-1 (provided by T. Wakita, National Institute of Infectious Diseases, Japan and C. Rice, Rockefeller University, NY) and SA13/JFH (provided by Jens Bukh, University of Copenhagen) were used to generate RNA as previously described (5). Briefly, RNA was transcribed using the RiboMAX express T7 kit (Promega) and electroporated into Huh-7.5 cells. Supernatants were collected at 72 and 96h post infection, pooled and stored at -80°C prior to infection of target cells. Cells were fixed with ice-cold methanol and infected cells identified by staining for NS5A with mAb9E10 and Alexa 488-conjugated anti-mouse IgG.

Quantification of HCV RNA. 2 x 10^5 HepG2.CD81 cells were seeded per well of 12 well plate and infected with HCV strain SA13/JFH for 8h, unbound virus was removed by sequential washes and the cells incubated at 37°C. Cellular RNA samples (Qiagen) were amplified for HCV RNA (Primer Design Ltd) in a single tube RT-PCR in accordance with the manufacturer's

guidelines (CellsDirect kit, Invitrogen) and fluorescence monitored in an ABI7500 real time PCR machine. In all reactions the housekeeping gene GAPDH was included as an internal endogenous control for amplification efficiency and for RNA quantification (primer-limited endogenous control, ABI).

Real-time PCR for phenotyping of activated macrophages and Kupffer cells

RNA was isolated from peripheral blood-derived macrophages and Kupffer cells using the RNeasy Mini Kit (Qiagen) according to the manufacturer's instructions. The isolated RNA was reversed transcribed using Superscript III (Invitrogen) with random primers (Promega) and dNTP mix (Invitrogen) on a Geneflow TC-512 thermal cycler. Real time PCR was performed in triplicate in 96 Multiwell plates (Roche) on a LightCycler 480 (Roche) machine. For each reaction 50ng of cDNA was used with QuantiFast SYBR Green PCR Master Mix (Qiagen) and the following QuantiTect Primer Assay Probes (Qiagen) used according to the manufacturer's instructions:

GAPDH (Hs_GAPDH_1_SG) Cat No: QT00079247

CD14 (Hs_CD14_1_SG) Cat No: QT00208817

CD16 (Hs_FCGR3A_1_SG) Cat No: QT00217252

CD163 (Hs_CD163_1_SG) Cat No: QT00074641

CD206 (Hs_MRC1_1_SG) Cat No: QT00012810

Analysis was performed using LightCycler 480 software version 1.5 (Roche) comparing relative fold change of mRNA to control.

Supplementary Figure Legends.

Supplementary Figure 1. Cytokine profile of macrophages and DCs stimulated with LPS. RNA from peripheral blood-derived macrophages and DCs were stimulated with 10μg/mL LPS (LPS M, LPS DC), 10ng/mL IL-4 (IL4 M) or unstimulated (US M) for 24h and RNA prepared. Cytokine mRNA levels were quantified using an Inflammatory Cytokines PCR array (SABiosciences, Sussex, UK).

Supplementary Figure 2. TNF-α mRNA and protein levels were quantified from macrophages stimulated with IL-4 (10ng/mL), LPS (10μg/mL) or unstimulated (US) or DCs from matching donors stimulated with LPS (DC LPS) for 24h. mRNA data was obtained from the array data shown in Supplementary Figure 1, and TNF-α protein levels were measured by ELISA.

References

1. Alabraba EB, Curbishley SM, Lai WK, Wigmore SJ, Adams DH, Afford SC. A new approach to isolation and culture of human Kupffer cells. J Immunol Methods 2007;326:139-144.
2. Tu Z, Bozorgzadeh A, Crispe IN, Orloff MS. The activation state of human intrahepatic lymphocytes. Clin Exp Immunol 2007;149:186-193.
3. Mee CJ, Harris HJ, Farquhar MJ, Wilson G, Reynolds G, Davis C, van ISC, et al. Polarization restricts hepatitis C virus entry into HepG2 hepatoma cells. J Virol 2009;83:6211-6221.
4. Theard D, Steiner M, Kalicharan D, Hoekstra D, van Ijzendoorn SC. Cell polarity development and protein trafficking in hepatocytes lacking E-cadherin/beta-catenin-based adherens junctions. Mol Biol Cell 2007;18:2313-2321.
5. Lindenbach BD, Evans MJ, Syder AJ, Wolk B, Tellinghuisen TL, Liu CC, Maruyama T, et al. Complete replication of hepatitis C virus in cell culture. Science 2005;309:623-626.

Supplementary Figure 1

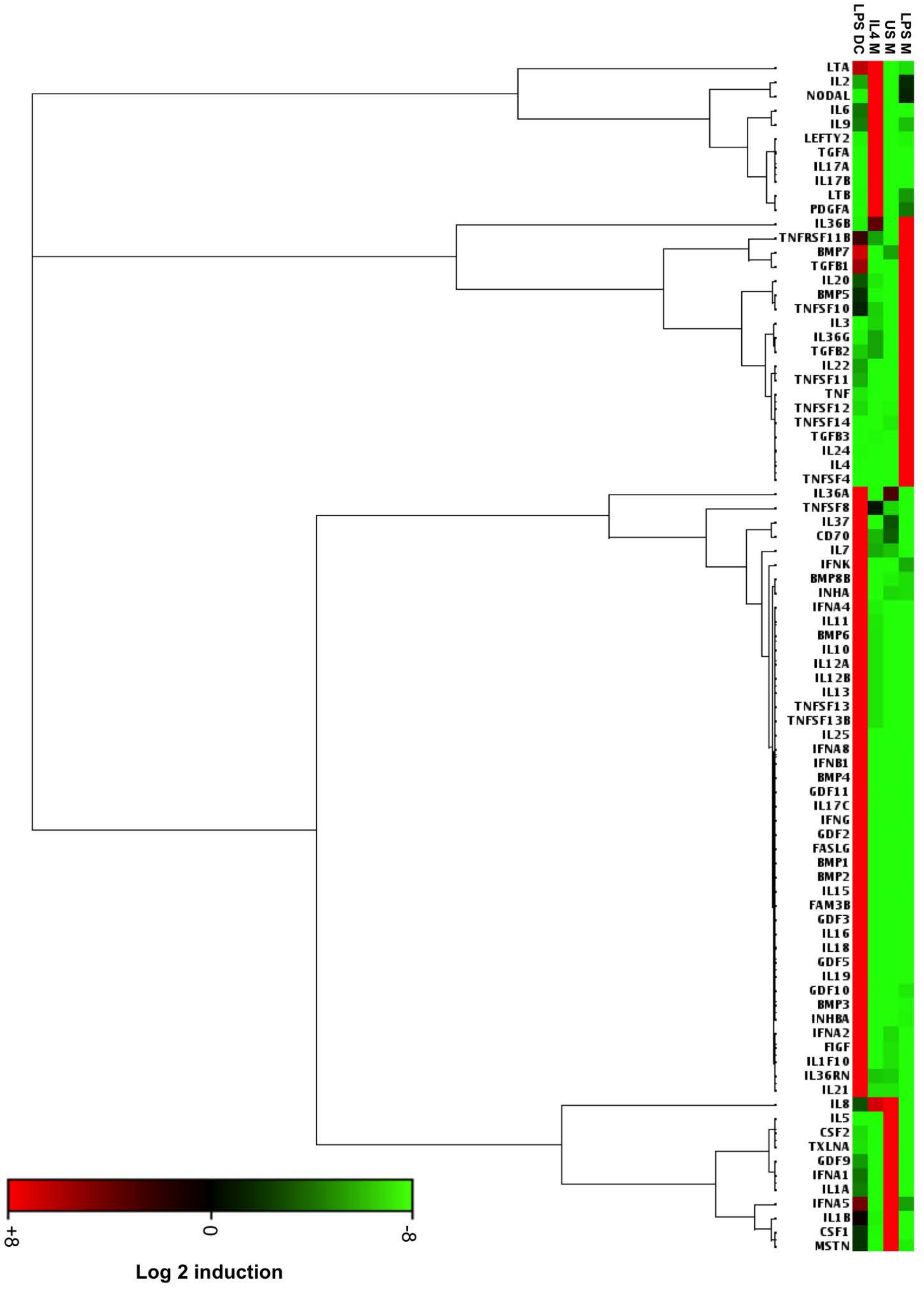

Supplementary Figure 2

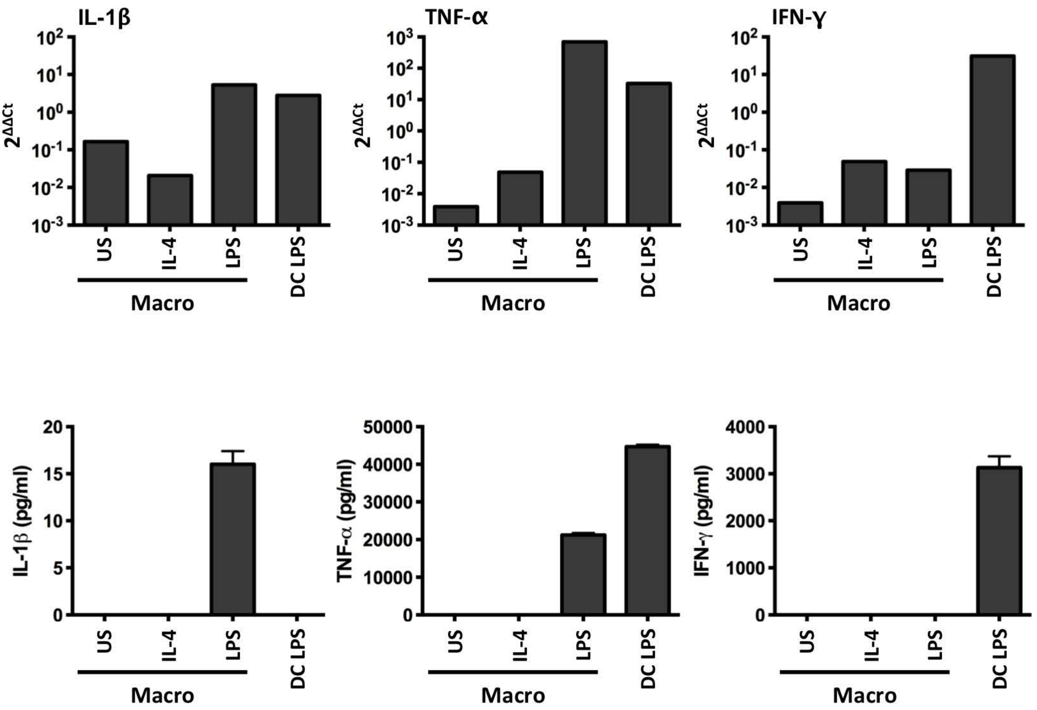

Supplement: Supplementary file 1 — Supplementary Information [file hep0059-1320-SD1.pdf]
